# StudyU: A Platform for Designing and Conducting Innovative Digital N-of-1 Trials

**DOI:** 10.2196/35884

**Published:** 2022-07-05

**Authors:** Stefan Konigorski, Sarah Wernicke, Tamara Slosarek, Alexander M Zenner, Nils Strelow, Darius F Ruether, Florian Henschel, Manisha Manaswini, Fabian Pottbäcker, Jonathan A Edelman, Babajide Owoyele, Matteo Danieletto, Eddye Golden, Micol Zweig, Girish N Nadkarni, Erwin Böttinger

**Affiliations:** 1 Digital Health Center Hasso Plattner Institute for Digital Engineering University of Potsdam Potsdam Germany; 2 Digital Engineering Faculty University of Potsdam Potsdam Germany; 3 Hasso Plattner Institute for Digital Health at Mount Sinai Icahn School of Medicine at Mount Sinai New York, NY United States; 4 Department of Medicine University Medical Center Hamburg-Eppendorf Hamburg Germany; 5 The Center for Advanced Design Studies Palo Alto, CA United States

**Keywords:** digital interventions, N-of-1 trial, SCED, single-case experimental design, web application, mobile application, app, digital health

## Abstract

N-of-1 trials are the gold standard study design to evaluate individual treatment effects and derive personalized treatment strategies. Digital tools have the potential to initiate a new era of N-of-1 trials in terms of scale and scope, but fully functional platforms are not yet available. Here, we present the open source StudyU platform, which includes the *StudyU Designer* and StudyU app. With the *StudyU Designer*, scientists are given a collaborative web application to digitally specify, publish, and conduct N-of-1 trials. The StudyU app is a smartphone app with innovative user-centric elements for participants to partake in trials published through the *StudyU Designer* to assess the effects of different interventions on their health. Thereby, the StudyU platform allows clinicians and researchers worldwide to easily design and conduct digital N-of-1 trials in a safe manner. We envision that StudyU can change the landscape of personalized treatments both for patients and healthy individuals, democratize and personalize evidence generation for self-optimization and medicine, and can be integrated in clinical practice.

## Introduction

A widespread aim in current medical research is to derive personalized treatment strategies, which are at the heart of treating every single patient with the best possible therapies. One motivation underlying this aim is that many drugs are only effective in up to 50% of patients [1-3]. Hence, treatment guidelines based on population-level randomized controlled trials (RCTs), which derive the best average treatment, may result in ineffective treatment or side effects in up to 50% of patients. As another characteristic of traditional RCTs, study participants only provide data for population-level analyses and do not profit from participation in the studies. Population-level RCTs are not meant to provide insights for individual participants. The gold standard design for evaluating individual-level treatment effects is prospective longitudinal N-of-1 trials [4], which are multi-crossover RCTs of sample size one [5,6]. That is, the study participant is administered the treatments of interest over time in accordance with a predefined setup of treatment length, duration, treatment blocks, and washout phases. In the literature, N-of-1 trials are sometimes used as a synonym for single-case experimental designs (SCEDs), mainly in the United Kingdom, but mostly used to describe a special case of SCED [7].

N-of-1 trials are well suited for studies when there is large interindividual heterogeneity in treatment effects, as well as when there are subpopulation groups or individuals with comorbidities of interest, who have been excluded from population-level RCTs. Important considerations relate to blinding, randomization, the typically arising correlation of measurements over time, and carryover effects of interventions, which can be approached either through the design of N-of-1 trials or in the subsequent statistical analysis. Series of N-of-1 trials can be aggregated to provide population-level estimates of treatment effects with similar efficiency to that of RCTs but require smaller numbers of participants [4,8,9]. Historically, there have been local implementations of N-of-1 trials in hospitals in the United States, Canada, and Australia [4,10]; series of articles on N-of-1 trials have been published in medical and epidemiological journals [11,12]; and networks on N-of-1 studies have been formed [13]. The advancements in digital technologies provide the potential to initiate a new era of N-of-1 trials in terms of scale and scope and have opened up new avenues to offer remote health care. In particular, performing N-of-1 trials digitally allows a seamless integration of trials in daily life, which can save time for participants and researchers since there is no need to visit a study center. This is especially important if daily measurements are collected. Extensive data can also be assessed passively to further reduce the burden on the participant if sensors are linked to a digital N-of-1 trial app. Finally, recruitment of participants can be simplified as participants from all over the world can participate in a trial, which is even more important for rare diseases where there are few potential participants [14].

Nonetheless, N-of-1 trials have not been integrated into mainstream clinical research or clinical practice. One of the underlying reasons might be that despite recently published guidelines [15-17], there are still considerations of the ethical framework when applying N-of-1 trials in clinical care [18]. Typically, series of N-of-1 trials designed with a specific research aim require ethics approval, while single N-of-1 trials with a clinical aim for a single patient do not require it. Often, however, this distinction is not clear. For example, let us consider a potential N-of-1 trial where physicians have the goal of finding out whether a particular drug is effective in off-label use for patients with chronic conditions such as chronic liver disease. The study setting involves patients treated in a specialized clinic department. In addition to treating the patients, the physicians in charge might be interested in knowing if the results are generalizable. In this situation, a series of N-of-1 trials can be designed, comparing standard care to standard care plus off-label drug use over different crossover periods. This example shows how N-of-1 trials can be woven into clinical practice and, as such, how their innovative design might be of high interest to physicians, if they can be performed easily. The latter point presents maybe the most important reason why N-of-1 trials have not been picked up more broadly since there is no platform available that allows for an easy and large-scale implementation of digital N-of-1 trials. As of now, conducting a digital N-of-1 trial generally necessitates the development of a new app.

Here, we present *StudyU*. *StudyU* provides an open-source, free, and easy-to-use platform with a study designer app for researchers, which allows easy design, customization, and implementation of N-of-1 trials, as well as a study participant app that allows participants to partake in these trials without having to set up user accounts. This serves to allow novel interactions among researchers, trials, and study participants.

## Design and Methods

### Related Work

A number of apps for conducting N-of-1 trials have been published. In order to attract physicians and researchers to design and conduct digital N-of-1 trials through a platform, the platform has to be accessible as easily as possible, should be able to implement interventions beyond mere symptom tracking, and, more generally, should allow designing studies flexibly for different interventions and different outcomes. Finally, analyzing the results in the app and providing the results back to the participant is an essential component to use the intrinsic patient-empowering potential of N-of-1 trials.

Table 1 presents, to the best of our knowledge, an overview of the most relevant apps that can be used to perform individual-level studies, particularly for N-of-1 trials. We want to note that it does not provide an exhaustive overview, and some other commercial platforms exist with limited publicly available information on their functionality; for example, the N of 1 platform by Digital Infuzion [19], which allows observational tracking of study participants. Furthermore, some apps have been developed, which focus on the cocreation of single N-of-1 trials by study participants themselves [20,21], which are not the focus here.

The Trialist app [22] provides results back to the participants and has been used in different N-of-1 trials but is currently not available for download and general use. The N1 app [23] had one study implemented for iOS users in the United States, which investigated the effects of caffeine and L-theanine on cognitive outcomes. It did not allow customization and further implementation of studies and is currently not available. Several apps provide functionalities for self-tracking and self-quantification but do not allow for an experimental evaluation of interventions (eg, mPower [24] and Parkinson mPower2 [24]). Of these apps, N1 and mPower are based on the Apple Research Kit. OpenClinica [25] allows creating and conducting studies but focuses on electronic data capture and data management and neither reports results to participants nor allows a collaborative creation of studies. QuantifyMe [26] is a platform that allows users to choose from a limited and prespecified set of interventions and outcomes to design a study without further customization possibilities. TummyTrials [27] and SleepCoacher [28] provide possibilities to choose from a set of specified interventions and investigate their effect on sleep and on food triggers in irritable bowel syndrome, respectively. Finally, PACO [29] and movisensXS [30,31] provide tools to design studies but are missing the main component of N-of-1 trials, in that the study app only gathers data—the results are not analyzed in the app and are not reported back to the study participant in the app. PACO has a further restriction that it is only available outside of the European Union and Switzerland. Furthermore, all of these mentioned platforms, except TummyTrials, require user accounts, which can create difficulties in terms of data privacy, especially if apps are planned to be used in different countries.

**Table 1:** Overview of existing apps and platforms that are suitable for gathering individual-level data. Some report the results of the conducted studies back to the user (column “Statistical evaluation of results”).

Name	App availability	Possible studies/ diseases	Platforms	Statistical evaluation of results	Customizable	Able to perform N-of-1 trials	Requires a user account	Link to the software
Trialist	No	Multiple options (for chronic pain only)	iOS, Android, or web	Yes	Limited options	Yes	Yes	N/A^a^
mPower	Only United States	1 (linked to Parkinson)	iOS	No	No	No	Yes	[32]
Parkinson mPower2	Yes	1 (linked to Parkinson)	iOS	No	No	No	Yes	[33]
PACO	Outside of the European Union and Switzerland	Flexible creation	iOS, Android, and web	No	Yes	Yes	Yes	[34]
movisensXS	Yes	Flexible	Android	No	Yes	Yes	Yes	[35]
OpenClinica	Yes	0	Web	No	Yes	Yes	Yes	[36]
N1	Only United States	1 (linked to cognitive health)	iOS	Yes	No	Yes	Yes	N/A
QuantifyMe	Source code only	4	Android	Yes	Limited options	Yes	Yes	[37]
TummyTrials	Source code only	4 (linked to irritable bowel syndrome)	iOS	Yes	Limited options	Yes	No	[38]
SleepCoacher	Yes	Multiple options (linked to sleep)	iOS and Android	Yes	Limited options	Yes	Yes	[39]
*StudyU*	Yes	Flexible creation	iOS, Android, and web	Yes	Yes	Yes	No	[40-42]

^a^N/A: not applicable.

### Vision

With *StudyU*, our goal is to attract more study participants and researchers to conduct and participate in N-of-1 trials by reducing the set-up process and implementation efforts. We envision that health scientists, medical researchers, and physicians worldwide can use it to collaboratively design and conduct N-of-1 trials. *StudyU* can therefore serve as a platform to contribute to open, transparent, and reproducible medical science by (1) making the study designs of different designed trials directly available to foster reproducibility and well-designed studies, and (2) making the anonymized data contributed by the study participants of the platform available for analysis to foster the generation of novel medical insights on health intervention effects at the individual and population levels. We envision enabling democratization and personalization for evidence generation in medicine and personal self-optimization.

### The StudyU Platform

**Figure 1:**
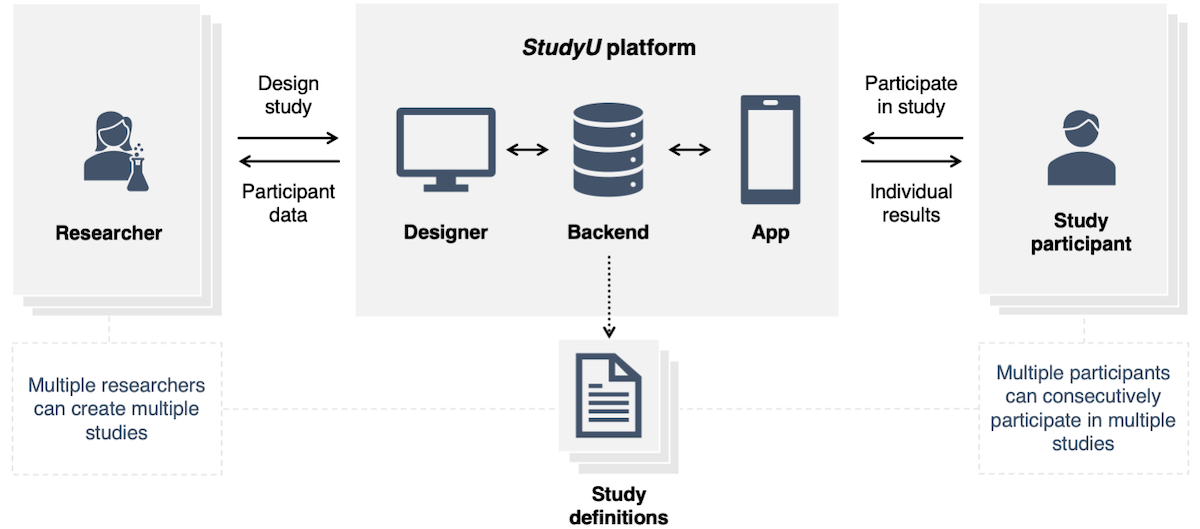
Architecture of the StudyU platform. Multiple researchers can collaboratively design and create studies and publish them. Then, study participants can partake in published studies. Study definitions and participant study data are stored in the backend.

The *StudyU* platform consists of 3 main parts, as illustrated in Figure 1 (see Supplementary Text 1 in Multimedia Appendix 1 for more details on the architecture):

1. the *StudyU Designer* web application for researchers,
2. the *StudyU* app for mobile devices, and
3. the backend where the participant data, study definitions, etc, are safely stored.

Designing and implementing a study with the *StudyU Designer* includes specifying the interventions (Multimedia Appendix 1, p19), eligibility criteria (Multimedia Appendix 1, p20), observations (Multimedia Appendix 1, p21), how they are scheduled, computing the results and displaying them to the participant (see *App*), and consent (Multimedia Appendix 1, p22). Such designed studies are then available to participants through the *StudyU* app. This user journey is described in more detail in Multimedia Appendix 1, Supplementary Text 2. The designer and the app are currently available in German and English, with apps in Spanish, French, and Korean planned in the near future. In the following sections, the technical setup and the main parts of *StudyU* are described.

#### Technical Setup and Use

The *StudyU* frontend applications are written in Flutter [43], an open-source, cross-platform user interface framework by Google based on the Dart programming language. With this, one single code base can be compiled to performant applications for multiple platforms: mobile, web, and desktop. Parse [44] is used as a backend, which is a platform that incorporates various functionalities such as object storage, user authentication, and push notifications. All components are organized and composed as Docker [45] containers for easy deployment. The source code for the *StudyU* platform is publicly and freely available on GitHub [40], and the *StudyU* app is available on Google Play and the Apple App Store [41,42]. For demonstration purposes, the backend is deployed on Back4App [46], and the frontend applications of the *StudyU Designer* and *StudyU* app are deployed on Google Cloud Run [47,48]. *StudyU* can also be deployed into any HIPAA (Health Insurance Portability and Accountability Act)–compliant and GDPR (General Data Protection Regulation)–compliant cloud system.

In the current implementation of *StudyU,* two choices can be made regarding how to use the platform, which provides flexibility to the needs of the researcher. First, *StudyU* can either be installed on one’s own separate server (or cloud) instance, or *StudyU* can be run and accessed on a central server operated by a third party. Second, studies can be designed and published individually or in collaboration with other researchers from other institutions. For collaborative design, studies can be accessed, edited, and saved by multiple researchers from multiple institutions. The studies can be accessed by multiple researchers at the same time, with the restriction that only one researcher can save data at the same time.

#### Study Model

**Figure 2:**
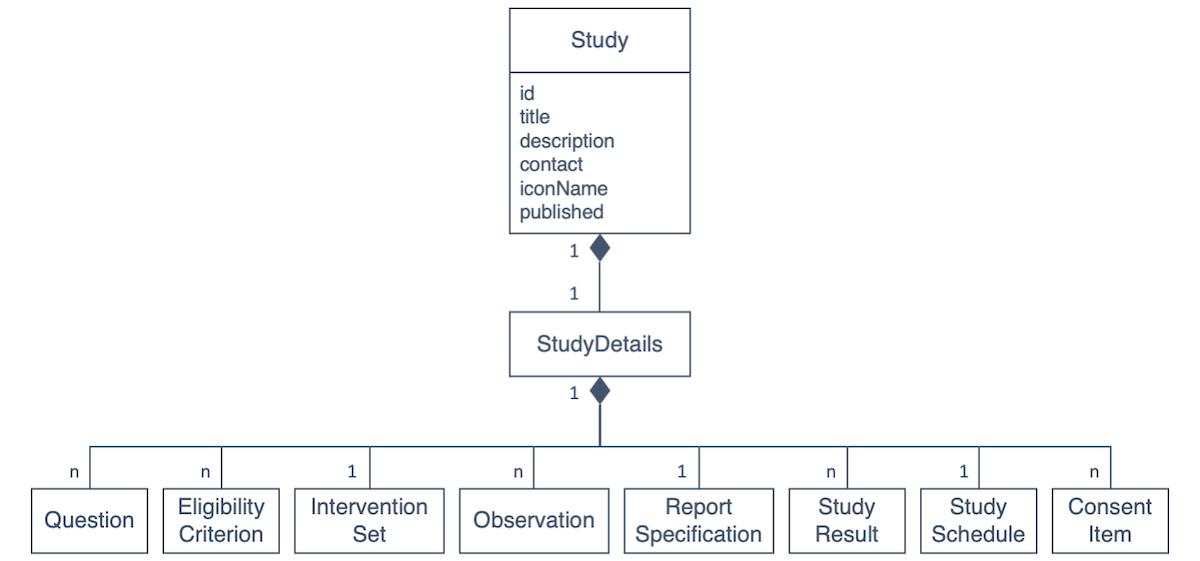
A simplified overview of the StudyU study model. The notation is based on the Unified Modeling Language class diagram notation, which defines properties of single classes in rectangles and associations between multiple classes as connections. The associations shown in this diagram with a filled diamond at one end mean that one class, for example, "Study," is composed of another class, in this case, "StudyDetails." Numbers shown at associations indicate how many instances of one class take part in this association, for example, n "Observation" objects can be associated with one "StudyDetails" object.

*StudyU* is based on a generic study representation, which is essential to dynamically support multiple studies. The representation encompasses study metadata and study details. The metadata of a study include basic information such as the title, a short description, and the researcher’s contact, including the name of the institutional review board (IRB) and protocol number. The study details contain all information that is needed to execute the study: eligibility questions and criteria, interventions, observations, specification of output and report data, schedule, and consent. All objects and relationships are serialized and stored in JavaScript Object Notation (JSON) format. The overall components of this study model are displayed in Figure 2 (see Multimedia Appendix 1, p23 and Supplementary Text 3 for more details).

The generic study model allows the design of many different N-of-1 trials in *StudyU*. This is illustrated in 2 example studies that are implemented in *StudyU*:

1. Investigation and comparison of the effect of any 2 of the following daily interventions on the intensity of chronic low back pain: willow bark tea, arnica balm, and warming pad
2. Investigation of the effect of any 2 of the following daily interventions on diffuse abdominal pain in irritable bowel syndrome: gluten-free diet, low-fiber diet, and fructose-free diet.

#### StudyU Designer

**Figure 3:**
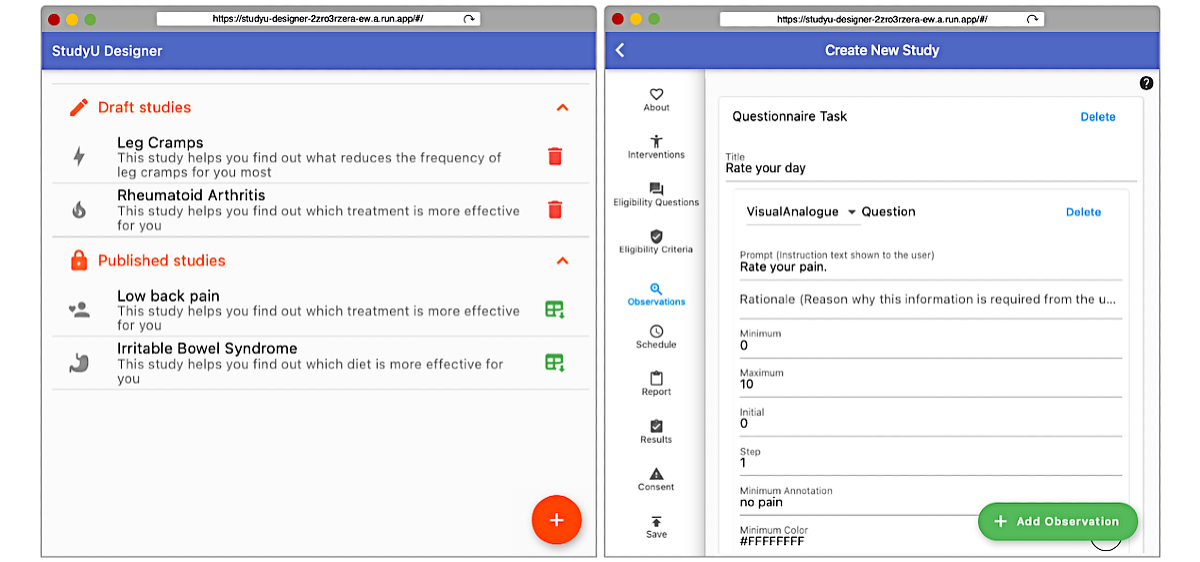
The dashboard of the *StudyU Designer* with drafted and published studies and an editor screen for observation definitions.

The *StudyU Designer* consists of 2 main components: the dashboard and the editor. The rationale behind this concept is to build a user-friendly tool for researchers, which provides a logical framework with all the necessary components to plan and conduct a study. Figure 3 shows the dashboard, which displays drafted studies and published studies. Once a study is published, it is available to users in the app and cannot be edited anymore in the designer. For published studies, researchers can download participant data in comma-separated values (CSV) format.

When adding a new study or editing a draft study, the editor leads through all study specifications as defined in the study model, such as interventions, observations, inclusion and exclusion criteria, consent, the format of the downloadable CSV file with study results, and the specification of reports shown to the user in the app. More editor examples and more details are shown in Multimedia Appendix 1, Supplementary Text 4. The sole responsibility for studies lies with the study designers, and in order to ensure the study participants’ appropriateness and safety of studies published in *StudyU*, the terms of use of *StudyU* prohibit misuse of the platform and require that researchers have conducted training on good clinical practice. Researchers have to include an IRB protocol number in the study metadata to assure participants of the adequacy of their study.

#### App

**Figure 4:**
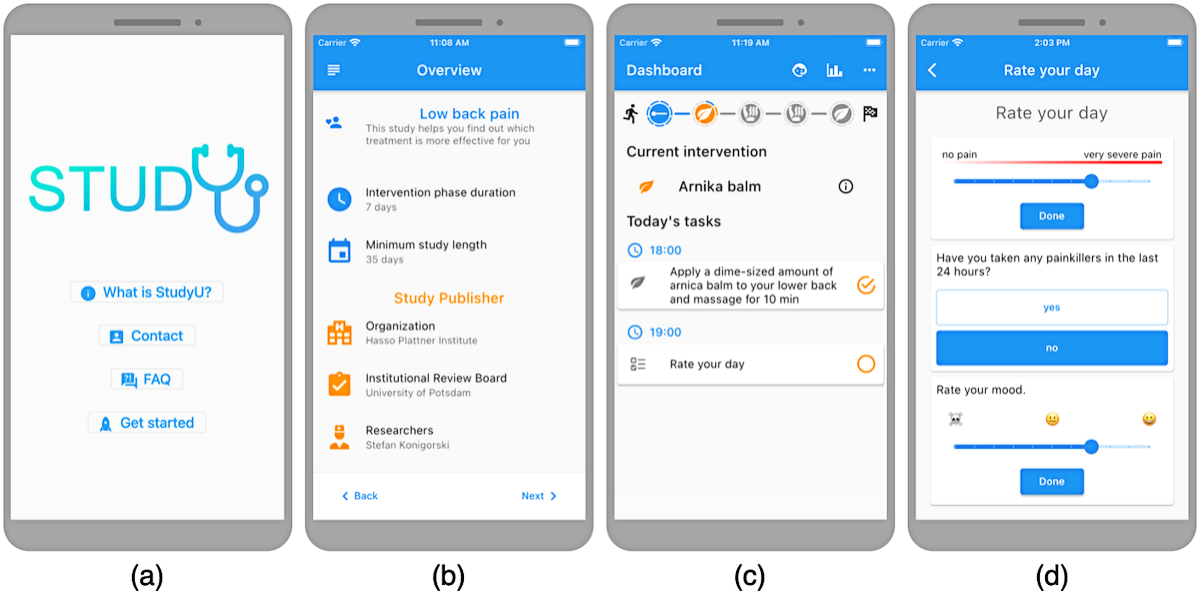
Initial screens of the StudyU app including the welcome screen, study overview screen, and daily screens.

The app enables users to participate in all studies that were created and published in the designer. This has the major advantage that participants do not have to download multiple apps for different studies but can partake in different studies through the same app and have an overview of all of them. After the welcome screen (Figure 4A), users can select which of the published studies they want to participate in. Before enrolling in one study, the study metadata are displayed in the study overview screen (Figure 4B). Then, users are led through the onboarding process with a validation of their eligibility, intervention selection, and declaration of consent. Finally, users arrive at the overview of daily tasks (Figure 4C), which contains the study progress bar and the daily tasks (Figure 4D). This is also the default screen users see when opening the app after the initial onboarding.

**Figure 5:**
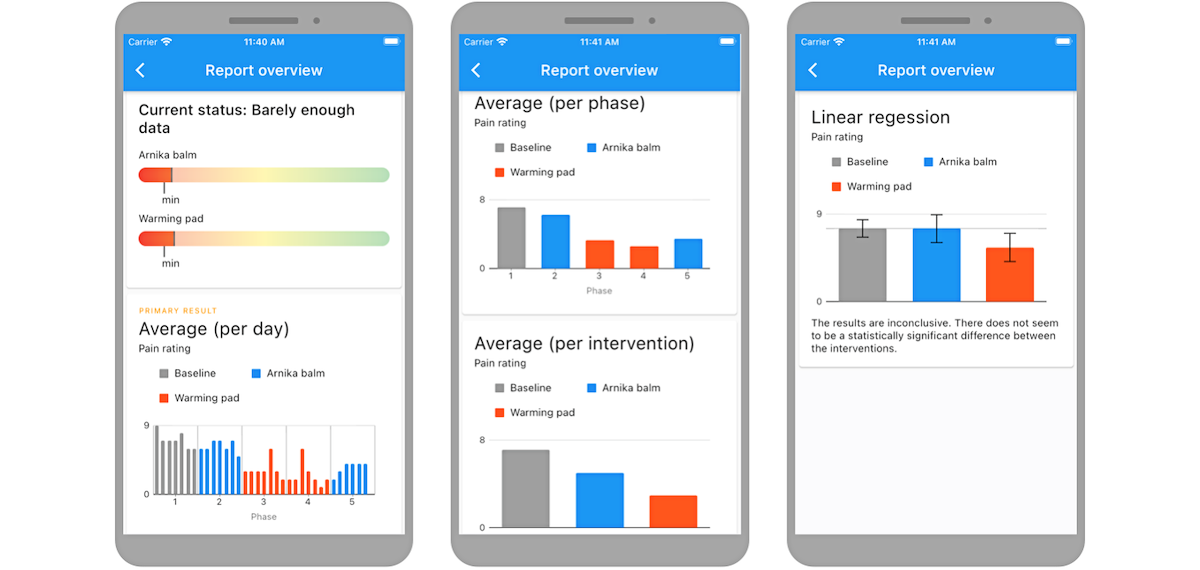
Examples of study reports. The power bar in the top-left panel indicates whether enough data were collected to observe an effect. Reports are displayed either as a linear regression report or as a report showing data aggregated by day, phase, or intervention.

A centerpiece of the *StudyU* app is the result visualization, which is illustrated in Figure 5. In order to ensure that no biases occur after having viewed the results, participants can only view them upon completion of a minimum study length specified by the researcher. For this purpose, a recommended study length is displayed to researchers in the designer, which should be calculated on the basis of a statistical sample size calculation. It should be noted that in the current demonstration of *StudyU* [48], the results are available from the first day in order to visualize the results. Through progress bars, the current status of the participant in the study is visualized to show how many more observations are needed; the effects of the interventions (if present) can be expected with the specified statistical power if the participants continue with the intervention at least until they reach the minimum study duration and report the measurements without missing data. In the study designer, different report types can be selected: (1) the visualization of a linear regression model that tests if the intervention has an effect on the outcome or (2) the report and explanation of individual results to the participant in bar charts. The definition of report types is implemented in an extensible way. More details are provided in Multimedia Appendix 1, Supplementary Text 5.

#### Data Processing

The studies carried out on the *StudyU* platform adhere to applicable ethical principles and international regulations, in particular, the GDPR (European Union) 2016/679 [49]. When the participant opens the app and accepts the terms of service, a new anonymous user account is created with a random ID that is assigned to the participant. Thus, no user profile is needed—which could be used to identify the participant—there is no log-in requirement for the app, and the participant does not need to create a password. The anonymous account is saved in the backend and on the device. Whenever the app is opened, the anonymous account also gets activated. If the participant completes a daily task, the results are stored inside the user study object and updated on the server. With this setup, there is no risk of data loss due to the study participant logging out of the app or forgetting a password. The only risk is losing the smartphone, in which case the anonymous link cannot be recovered. There is the possibility to opt out of the study, which deletes the unfinished study and the local storage and reference to it. The participant can also choose for his/her data to be deleted locally and on the server. These are some important principles of good clinical practice, and we further require every researcher using the *StudyU Designer* explicitly to have followed training in good clinical practice.

The legal basis for processing the study data is the consent provided by the participant via the researcher-defined consent form. Researchers can link and analyze data in different ways in the backend using random user IDs but cannot link them to specific participants. The setup of *StudyU* does not collect identifiable information, and we also discourage researchers from assessing information which includes participant-identifiable data in their designed studies. We anticipate that this setup without user accounts will satisfy the regulations and data security standards in most countries, allowing a broad use of *StudyU*.

## Discussion

Here, we have presented the *StudyU* platform, which allows researchers to undertake an easy design, customization, and implementation of N-of-1 trials and allows individuals to participate in those trials without having to set up user accounts. Through the *StudyU Designer*, researchers can collaboratively design trials. *StudyU* is available open-source for iOS, Android, and the web, is free to use, and provides anonymized data entry, which prevents tracing back the data to the participants. It allows conducting the entire study process digitally: study design, participant recruitment, inclusion and exclusion of participants through the study app, automatically analyzing the individual data in the app and reporting the results back to the participant, and saving the data in the secure backend so that researchers can analyze it further and aggregate it across N-of-1 trials. As further innovative concepts, we provide electronic consent and the possibility for the study participants to view their progress through the study on a progress bar. With these features, *StudyU* is currently the only available platform that allows flexibility to the N-of-1 trial design and the capacity to conduct them completely digitally. All other existing apps have limitations in the platforms they support, the possibility and customizability to design individual trials, the freedom to use the app without having to set up a user account, and the automated in-app statistical analysis to provide the results back to the participant.

As participants likely start the trial with high intrinsic motivation and high expectations of getting insights into their health, it is critical that they are not disappointed and drop out of the trial. The progress bar keeps the participant informed when they have reached the targeted study length and when they can view a statistical evaluation of their results. We expect that it can also encourage them to continue the study for a longer time before viewing the results in order to estimate more precise treatment effects, thereby extending the classical statistical power–based sample size calculation. With this, we envision that participants understand the value of long-term participation in the study and stay motivated for a longer time so that dropout rates can be decreased, thus mixing elements of extrinsic motivation to intrinsic motivation [50]. Regarding the use of progress bars, there is some conflicting evidence in the literature [51,52] regarding whether they have a positive effect on increasing adherence, with some suggestions that only specific types of progress bars (ie, fast-to-slow presentation) are beneficial. For this reason, we propose a new approach of the progress bar in future iterations of *StudyU* to offer participants the chance to look at the results at any time if they want, with the caveat that their results will only be statistically evaluated once to avoid biased results. We expect positive effects on study adherence of such a design, similar to the endowed progress effect [53].

Using the *StudyU* platform, N-of-1 trials can be designed not only to study the effect of many different health interventions and lifestyle factors on health outcomes in rare diseases and chronic diseases, but also to evaluate the effectiveness of digital health apps. N-of-1 trials can evaluate the effect of health interventions truly in the real-world setting. Especially with the ongoing COVID-19 pandemic highlighting the importance of remote and digital medicine, evaluation and digital integration into the home environment are of high value. While fully digital trials in the home environment can provide challenges for N-of-1 trials owing to possible carryover or confounding effects that have to be considered in the automated analysis, the challenges can be addressed through the implementation of more advanced statistical and machine learning methods. In fact, recent years have seen an unprecedented development of deep learning methods for estimating the individual-level effects of health interventions from population-level studies and to predict individual disease trajectories and individual treatment effects [54-58]. These methods are often based on nontestable assumptions, require large data sets, and have limitations in their interpretability of individual treatment effects in complex causal graphs. Combining them with the design advantages of N-of-1 trials can help derive fully automated analyses of complex real-life trials.

Two important considerations in N-of-1 trials are randomization and blinding to ensure that unbiased estimates of causal effects can be obtained. Randomizing study designs in a within-persons (eg, the order of treatment A and B within each cycle) and a between-persons manner can be implemented in *StudyU* if desired, but it should be considered that a deterministic sequence might be able to counterbalance specific time-confounding effects for a given participant, while a randomized sequence can achieve this on average. Blinding can occur on 2 levels: blinding researchers to treatment allocation and blinding study participants to treatment allocation. Researchers can be blinded in *StudyU* by incorporating another person who controls the allocation in the design of the trial. Blinding of study participants with respect to which interventions they are currently following is not possible in many digital N-of-1 trials, as, for example, drinking tea or using a warming patch are visibly different. Blinding would have to be achieved with help of a researcher, physician, or third person and can be implemented in *StudyU* by naming interventions anonymized A and B and providing, for example, similar looking pills for A and B. Such blinding can prevent biases that might arise from the participants during the trial. However, this has to be balanced with the aim of N-of-1 trials to benefit and empower the participant. More importantly, it should be remembered that any conclusion that intervention A works better than B for a given participant also holds for any nonblinded trial—we only do not know the extent to which this effect was due to the intervention or accompanying beliefs. However, the participant might not care why the intervention worked but might rather care about the fact that it worked.

We plan to include several extensions in *StudyU* in the future. First, for the study designer, we plan to add more features encouraging the collaboration on study designs. Setting up a database of interested researchers, clinicians, and institutions can help search for partners in designing and conducting the studies. In the current version of *StudyU*, all studies are, by default, public to enhance full collaboration and allow for open-access study development. We are working on a more fine-grained collaboration platform, which allows the researcher to make both the creation and conducting of studies fully public or private to a selected group of collaborators and selected group of invited study participants. As a second new feature in *StudyU*, we will include the possibility to link sensor-based data to measure health outcomes and covariates and also allow the integration of other digital health apps in *StudyU*. Third, we will provide the possibility to design adaptive trials, for example, including elements from microrandomized trials and just-in-time adaptive interventions [59-61]. Fourth, we plan to implement a more elaborate progress bar visualizing the study progress of the participants. The current progress bar is based on the study duration and number of past measurements, but a more exact measurement would be to focus on the number of nonmissing measurements. This feature can be added by including an automated check for the validity and completeness of the recorded data and feeding it back into the progress bar. Fifth, we plan to integrate more complex statistical and machine learning methods in the study app so that complex individual-level treatment effects of potentially time-varying treatments and time-varying confounders can be included in the modeling and result reporting to the individuals. Currently, only linear regression and *t* tests are implemented in *StudyU*. They provide simple models with easily interpretable results and have been shown to provide efficient and robust treatment effect estimates even when autocorrelation and time trends are present [62]. Nonetheless, implementing more complex statistical models such as Bayesian mixed models or G-estimation will allow a more fine-grained and powerful analysis. Finally, we are working on the development of user-centric N-of-1 trials designed by the study participants themselves and are excited to integrate these study designs as well as the study results into *StudyU* [20]. We envision that placing a higher focus on the cocreation of trials with participants can be very important to increase adherence to the trial, especially for long-term experiments, which is not straightforward as shown in other studies [21,63]. Furthermore, fully cocreated trials, where the participant defines what he/she wants to evaluate, might have a higher chance of exerting an actual effect on health behavior change. It would be interesting to embed such trials into models of health behavior change, such as the one by Prochaska et al [64], and think about which elements can map to each stage of precontemplation, contemplation, preparation, action, maintenance, and termination. Building on this, linking N-of-1 trials further to electronic health records in the future has the potential to connect N-of-1 trials into clinical care and clinical workflows and can further enhance the integration of medical research and clinical practice.

## Appendix

Multimedia Appendix 1 Supplementary text and figures.

## Abbreviations

CSV: comma-separated values
GDPR: General Data Protection Regulation
HIPAA: Health Insurance Portability and Accountability Act
IRB: institutional review board
JSON: JavaScript Object Notation
RCT: randomized controlled trial
SCED: single-case experimental design
