# Supplementary material for: StudyU: A Platform for Designing and Conducting Innovative Digital N-of-1 Trials
Source: J Med Internet Res. 2022 Jul 5;24(7):e35884. doi: 10.2196/35884 (PMC9297132; doi:10.2196/35884)

StudyU: a platform for designing and conducting innovative digital N-of-1 trials
Supplementary Material

Supplementary Text 1: Architecture of *StudyU* **3**
- Overview 3
- Flutter 3
- Parse Server Backend 3
- *StudyU* App 4
- *StudyU* Designer 4
- Core Package 5

Supplementary Text 2: User Journey **6**

Supplementary Text 3: Study Model **7**
- Overview 7
- Elements and Aspects 7
- Eligibility Questions 8
- Reports 8

Supplementary Text 4: *StudyU* Designer **9**
- Motivation 9
- Components 9
  - Dashboard 9
  - Editor 9
- Features 10
  - Inline Editing & Icon Picker 10
  - Type Changing 11
  - Nested Expressions 11
  - Slider Questions 12

Supplementary Text 5: *StudyU* app **13**
- App Onboarding 13
  - Welcome Screen 13
  - About Screen 13
  - Terms and Conditions Screen 13
- Study Onboarding 13
  - Study Selection Screen 13
  - Study Overview Screen 14
  - Eligibility Check Screen 14
  - Intervention Selection Screen 14

Journey Overview Screen 15
Consent Screen 15
Study Participation 15
Dashboard Screen 16
Task Screen 16
Contact Screen 17
Report History Screen 17
Report Overview Screen 17
Performance Screen 18
Settings Screen 18
Supplementary Figures **19**

Supplementary Text 1: Architecture of *StudyU*

In the following, we give more details on the architecture of *StudyU*.

Overview

Our system consists of three parts. First, the *StudyU* designer web application for researchers to create studies. Second, the *StudyU* App for mobile devices for participants to conduct the study. Finally, a backend including a database to save the study model, to provide it to the participants and to save the progress of the participants.

The workflow of the system is as follows:

1. The researcher creates and publishes a new study using the designer.
2. The participant selects the study in the App.
3. The participant completes the daily intervention and observation tasks.
4. When the participant finished the study, they can see which intervention improved the outcome for them.
5. The researcher can download the anonymized data of all participants as CSV and use it for further analysis.

Flutter

Both the App and Designer are built in Flutter. Flutter is a cross-platform mobile application framework. It is being built by Google using their language Dart. The first stable release was published in December 2018. Besides Android and iOS, it supports running as a web application.

Our decision was led by multiple requirements. Flutter allows building an app for both iOS and Android, without developing two separate apps. Flutter has a lot of ready-made components and functionality, developed first party by Google. They provide components in Material and Cupertino design. Flutter also has performance advantages compared to, for example, React Native. Flutter renders all components using Open Graphics Library (OpenGL). This gives it an advantage in performance.

We decided to also use Flutter for the *StudyU* designer, but focused on building a web app. Using Flutter Web has the advantage of reusing parts of the designer, especially the study model classes. Multi-language support and integration with our backend were also reused. We extracted the code into a separate package called core.

Parse Server Backend

As backend, we used Parse Platform. It provides an easy setup and integration with our app and allows storing studies and user progress. It is an open-source backend focused towards building mobile applications. It was first developed as a service, later bought by Facebook and open-sourced. Parse supports object and file storage (uses MongoDB as a database), user

authentication, push notifications and has a dashboard. We used the Parse Flutter Software Development Kit (SDK), which is an almost complete implementation of the Parse API.

We built a Parse setup with docker-compose, which can be run on every machine with Docker installed. The Parse backend stores all data produced by the applications. First when the *StudyU* App is started, an anonymous user is created and used to associate study participation to that user. The user is stored on the device and does not carry any information of the participant. We also store the studies, created by the designer, split into tables *Study* (basic information) and *StudyDetails* (complete study model). Each time a user starts a new study, a new *UserStudy* object is created. This object holds all information that is needed to run the study for that user. It saves study information such as name and description, but also properties set by the user, such as the selected intervention. We duplicate this data to ensure that changes to the study will not interfere with already running studies. Every time a user completes a task, its results are stored on the server. This allows the researcher to always check how the running studies are doing and how many participants the study has. The researcher can also download and analyze anonymized data of participants. Parse supports sending push notifications to the phones. This could be used to – anonymously – encourage users to finish a study or make them aware of certain events without them needing to open the app.

StudyU App

We built the *StudyU* App to run primarily on iOS and Android, but it can also run on the web. The app has multiple instances where it interacts with the Parse server or the internal store. First when the users open the app and accept the terms of service, a new anonymous user account is created. It is saved in the backend and on the device. Each time from then on when the app is opened, we have an active user account until the user deletes the app or deletes its data. After the terms are accepted, the app fetches all published studies from the backend. When the user then selected a study and made their intervention choices, the app creates a new *UserStudy* instance and copies all data needed to run the study from the *Study* and *StudyDetails* instance. Additionally, a reference to the newly created *UserStudy* object is saved inside the local storage. Each time the app is started, we check if a *UserStudy* is present in the local storage and fetch the corresponding object from the backend with it.

Each time the user completes a daily task, the result is saved inside the *UserStudy* object and updated on the server. The app also has the option to opt-out of the study, which deletes the unfinished study and the local storage reference to it and sends the user back to study selection. The user can also press on delete, which deletes the user locally and on the server, but does not delete the current study. This will be changed in the future, for consistency with the opt-out method.

StudyU Designer

The *StudyU* Designer was built using Flutter, with a focus on a web version of the application. The *StudyU* Designer creates the studies and saves them on Parse. Studies have a published attribute. An unpublished study is not seen in the app and can still be edited. Once published the study is seen in the app, but cannot be changed in the *StudyU* Designer anymore. The

StudyU Designer also supports Android and iOS, which will be added in future versions of *StudyU*.

Core Package

Since both the App and the Designer use Flutter and Dart, we split out common functionality into a separate package called core. The package is imported both by the App and Designer and holds all the model classes and some other functionality used by both apps. It is a great starting point to build an additional application using the same study model, as all model classes are contained in the package. Furthermore, we could extract more shared code, even User Interface (UI) components, which are needed in both.

Supplementary Text 2: User Journey

In this section, we provide an overview on the main concepts of the *StudyU* platform. For each step, the view of the researcher who designs the study and the user who participates in the study are shown.

In the interventions section (Supplementary Figure 1), the researcher is able to specify treatments or therapies that will be applied in the study over time at specified time intervals, by using text and icons. These interventions are safely stored in the backend, and then the study participant can choose from them for the study. Details are displayed under the information icon and also the involved daily tasks are shown here. It is not possible to select more than two interventions per study.

In N-of-1 trials and clinical trials in general, the definition of inclusion and exclusion criteria for participation in the study is of particular importance. This can be done in the designer through the two sections ‘Eligibility Question’ and ‘Eligibility Criteria’ (Supplementary Figure 2). The eligibility criteria can be flexibly set up based on the answers to one or multiple questions. For the App, an explanation can be provided to the participant explaining the exclusion criteria.

Supplementary Figure 3 shows how observations can be defined in *StudyU*, i.e., all primary and secondary outcomes, as well as further covariates of interest such as lifestyle or environmental factors. The current version supports two task types: tasks that can be checked upon completion and questionnaire tasks. The modularity of the app also allows the extension of further task types. For questionnaire tasks, different question types such as multiple-choice questions or logical statements can be selected as well as rating scale questions. Annotated gradient colors, appealing texts and emojis help to enhance the user experience. After completing this section, the researcher can specify the schedule. The study length can be defined under ‘StudySchedule’ where the desired number of cycles, duration of phases in days and a baseline phase can be defined. Further, the sequence of interventions can be specified as alternating, counterbalanced or randomized. In the *StudyU* app, the participant gets an overview of the final study schedule with a detailed study description and information about the responsible researchers. The start date of the trial is shown for each study phase and the last entry in the list marks the final trial session after which the study results can be viewed.

As we value a good understanding of study consent by the study participant of great importance, we aimed to define and convey the consent display in a comprehensible and appealing way using modular, topic-specific boxes (see Supplementary Figure 4). The rationale behind the design of the screens is to make the consent process stripped-down and as attractive as possible. This is realized through icons and animations that can be added to the boxes. Study participants can trace which modules they already read through the changing colors of already opened boxes. At the end of the consent process the participant agrees or refuses to take part in the study. The content can also be saved on the participant’s device.

Supplementary Text 3: Study Model

In the following, we give more details on the study model, which is shown in Supplementary Figure 5.

Overview

One desired goal of the project was development of a platform that can handle multiple different studies. This raises the question of how to distribute the studies to the user. The studies should be executed using a mobile app available in the respective app store of the device. However, if an update would be required for every new study, the deployment of a study would take a very long time due to review processes in the app stores. Hence, the app should be capable of executing new studies without the need of updates. To solve this problem, we treat study designs as files that are stored on a server. When a researcher designs a new study, the design is made available to all users of the App. A user can then select the study to start participating in it and gets also the study contact information displayed. The design is downloaded by the app and executed according to the ideas of the researchers. Once the user finishes the study, they can select another study to participate in.

To allow for the representation of studies as files, we defined a data model for N-of-1 trials which covers all common elements of N-of-1 trials. This model consists of a set of objects and their relationships and is serialized using JavaScript Object Notation (JSON). It is split into two parts, metadata and details. The metadata of a study contains its title, a description, the research group and other aspects that are shown when the user looks at the list of available studies. The details of a study contain the study description and all information that is needed to execute it. These details are further separated into multiple aspects which map to the common elements of N-of-1 trials.

Elements and Aspects

An N-of-1 trial consists of interventions and observations. In the model, the interventions are stored as an *InterventionSet* which stores a list of *Intervention* objects. The observations are represented as a list of *Observation* objects. The desired schedule of the interventions in the study is stored as a *StudySchedule* object. This contains the desired number of cycles and the duration of a phase in days. Further, the inclusion of a baseline phase can be configured. From these values, the total duration of the study can be calculated. The sequence of interventions can be specified as alternating, counterbalanced, or randomized.

The exclusion criteria are split into two elements, an eligibility questions and a set of eligibility criteria. The eligibility questions consist of a list of *Question* objects. The eligibility criteria are Boolean expressions that reference the answers to questions. If any criterion evaluates to false, the user is excluded from the study and provided the reason specified in the model. If all criteria evaluate to true, the user is eligible to participate in the study. To provide facilities for informed consent, the consent process is broken down into multiple items. These items might include risks of the study, privacy considerations, and funding information. Each of these items is represented as a *ConsentItem*.

To provide understandable results to the user and facilitate further analysis by researchers, the outcomes observed during the study are processed in two ways. First, a visual and easily comprehensible report is generated for the user based on a *ReportSpecification*. Second, the aggregated and anonymized results can be exported by researchers for the analysis in other tools as specified by a set of *StudyResult* objects. We will further examine two aspects in detail, the *Question* objects and the *ReportSpecification*.

Eligibility Questions

As stated above, an eligibility questions item consists of a list of *Question* objects. When eligibility questions are presented to a user, the questions are presented in order. There are four types of questions to cover different types of data to be collected. First, a *BooleanQuestion* covers yes or no questions such as “Have you had back pain in the last 12 weeks?”. Second, a *ChoiceQuestion* provides single-choice or multiple-choice questions which can be used to inquire about gender or present allergies. Finally, a *VisualAnalogueQuestion* and *AnnotatedScaleQuestion* inquire numerical values in a range via a slider. The difference between the last two types is the scale that is displayed next to the slider.

The answer to a question is stored in an *Answer* object. Based on a set of answers for eligibility questions, *Expression* objects are used to represent Boolean expressions for the eligibility questions. As stated previously, these expressions are used to determine the eligibility of the user. Further, they can be used to make some questions optional. One example for this is the exclusion of the question “Are you pregnant?” if the user already specified that they are male. If a question is excluded because of such a condition, a default value is used instead.

Reports

As stated previously, interventions and observations consist of tasks. Each type of *Task* can produce results which are stored every time the task is performed. The type of results includes whether the user completed a simple task or answers to a daily questionnaire. These results are associated with the intervention that was active at the time for the purpose of analysis.

For the visual presentation of results to the user, a *ReportSpecification* is used as a blueprint. A report consists of at least one primary *ReportSection* and optional multiple secondary sections. There can be different types of sections that reference the results generated over the course of the study. One simple example for a section is the *AverageSection*. This should present a single observed outcome averaged over a configurable time frame (either per day, per phase, or per intervention).

As there can be many different kinds of tasks, the format of results of these tasks can vary widely. To provide a configurable method to reference observed outcomes of different tasks, *DataReference* objects are used. These data reference objects specify a type of data they refer to (e. g. numerical values). A reference consists of a task identifier and a property identifier. A task might expose only one property (the user completed the task) or multiple properties (the answers to a questionnaire). When resolving a data reference to generate a report, the type of the property is checked and a list of values with accompanying timestamps is exported. The values can then be used by the report section to present the data to the user.

Supplementary Text 4: *StudyU* Designer

In this section we describe the *StudyU* Designer, the app that lets researchers design and publish studies. We motivate why this app is needed and look at its components, including a more detailed description of certain features it provides along with some of the highlights of its implementation.

Motivation

As introduced in the previous section, the study model we designed generalizes N-of-1 trials and offers the researchers a lot of flexibility to create their own studies just the way they envision them. However, the following question arises: How can the researchers incorporate their ideas on how the study they want to conduct should look like? And how can they deploy the final study on our platform? One solution would be to simply allow the researchers to upload a study according to the schema of our Study Model and in the JSON file format that our platform uses to store the studies. With this a validation check of the JSON against our study model would suffice. This approach shifts a large amount of work to the researchers, and would require more technical expertise. Instead, we wanted to achieve the goal of building an easy-to-use app for the researchers, that provides a logical order of defining the different parts of a study as well as the required building blocks that can be configured with relative ease. Our hypothesis is that by reducing the friction of the setup process, ultimately more researchers will be willing to conduct N-of-1 trials using our platform. This in turn makes the *StudyU* App more attractive to its users, as it provides more studies to participate in.

Components

The Designer can be divided into two main components, the dashboard and the editor.

Dashboard

The dashboard shows the researcher two lists of studies: draft studies, which are studies that are still being worked on, and published studies, which are studies that users can participate in. Draft studies can still be deleted by the researcher. For published studies, the results can be downloaded.

Editor

The editor allows the researchers to specify the different aspects of their study according to our previously introduced study model. The editor is split further into sections that allow the user to focus on each aspect separately and to have a better overview. Also, we have arranged these sections in an order that represents a logical way of approaching the setup of a study. However, the order is not mandatory and the researchers can switch between the sections as they please. In the following we will introduce the sections briefly in the same order as they are presented in the Designer.

a. Meta Data: This section allows the researcher to edit the study title, description and select a descriptive icon to represent the study.

b. Interventions: Here the researcher can define the different interventions that are offered to the user, including the tasks each intervention comprises and how these tasks are to be scheduled. We put this section second, just after the Meta Data section, as the interventions are the main characteristic of a study and we expect them to impact the other setup decisions.
c. Eligibility Questions: In the eligibility questions, the questions the user needs to answer before participating in the study are added. The researcher can specify Boolean questions as well as single and multiple-choice questions.
d. Eligibility Criteria: Using the questions defined in the section before, the researcher can now define the eligibility criteria by specifying what answers lead to an exclusion, together with a reasoning for the users on why they are excluded. This section can only be edited once the eligibility questionnaire above contains at least one question.
e. Observations: In this section the researcher defines the observations the user has to report on and how they are to be scheduled. In the current version the user can be asked to answer different questions. Additionally, to the Boolean and choice questions already mentioned, the researcher can also set up slider questions.
f. Schedule: The schedule section is used to edit the study's schedule information, including the number of cycles, phase duration, whether or not a baseline phase should be included and the sequence of the interventions. Different interventions have different impacts, e.g., a longer washout period or a longer time until an effect can be observed.
g. Report: Here the researchers define the setup of the report the user receives. They can add and configure the different report sections.
h. Results: This section allows specifying which observation results the researchers want to include in their data export.
i. Consent: In the consent section, the researcher can set up the study-specific consent form in a modular way. Placing the consent section last allows the researchers to take all their previous settings into account. For instance, the researcher might be using all, a selected few or none of the observation results, each option impacting the content of the consent.
j. Publish: The final section offers the researcher the options to save the study as a draft or to publish it. If saved as draft, the researcher can continue editing the study at a later point in time before eventually publishing it. When the researcher wants to publish the study, it is first validated, to check whether all necessary aspects are specified. Once published it is available to the users of the *StudyU* App.

Features

In the following, we highlight some of the features we included to make the Designer easier to use.

Inline Editing & Icon Picker

As can be seen in the actively edited title field below, the researcher just needs to click into a field to edit its value, instead of having to open an edit model or having to navigate to an edit page.

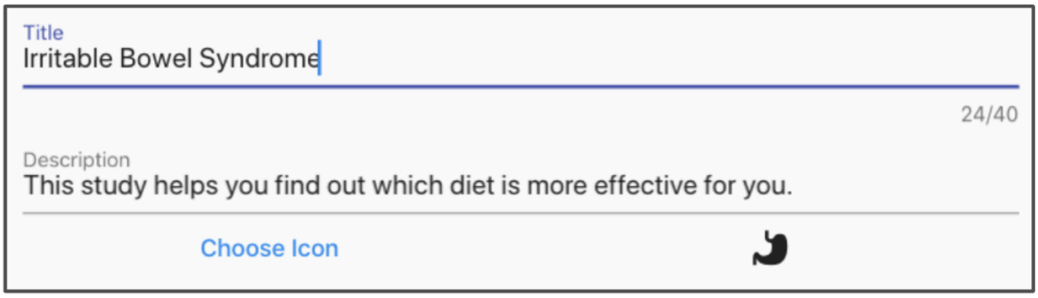

We offer the researcher to select icons, for the study itself, its interventions and the consent items as visual clues for the user.

Type Changing

The study model offers multiple types for certain elements of a study. As an example, a question can be a Boolean, choice or slider question. This poses the question how the researcher can easily choose the desired type while setting up the study. One option would be to offer multiple add buttons, one for each type. However, that would make the interface more complicated. Instead, we only offer one add button and initially add the most basic type of an element, in case of the question element that is a Boolean question. Then the researcher can easily switch the type with a drop-down button. The type can also be switched after the element is already edited. When changing the type, we keep the values for shared attributes between the old and new type of an element, such as the prompt field shown below. This saves the inconvenience of having to type things again.

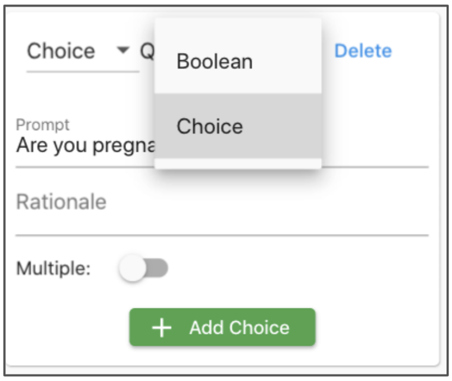

Before

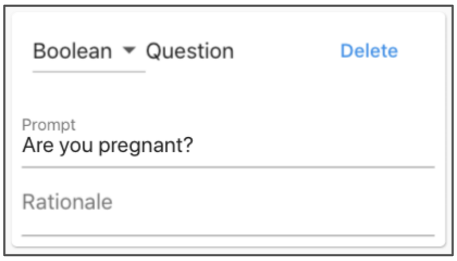

After

Nested Expressions

When defining the eligibility criteria, the researcher defines via expressions what eligibility answers are required for the user to be eligible. By nesting a value expression inside a not expression, a negation can be achieved. The nesting is also supported visually in the designer as shown below. In the shown example the criterion defines that a participant must answer the “Are you pregnant?” question with “no” to be eligible.

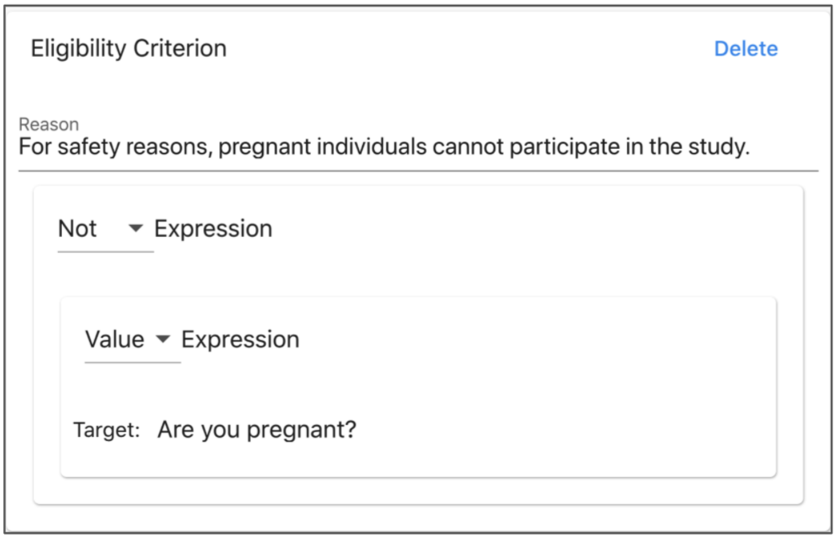

Slider Questions

One of the best examples of the amount of flexibility our study model and therefore the *StudyU* Designer offers the researcher is the configuration of slider questions. The researcher can define the minimum, maximum and initial value, as well as the step size of the slider. In case of a visual analogue question, annotations for the minimum and maximum value can be added and even the color gradient is customizable. The figure below shows the section of the editor for these values in the designer and how the question is displayed to the user in the app. It illustrates that the color gradient can be configured to underline the intention of the question, in this case choosing between the most positive and most negative option by sliding along a white to red gradient.

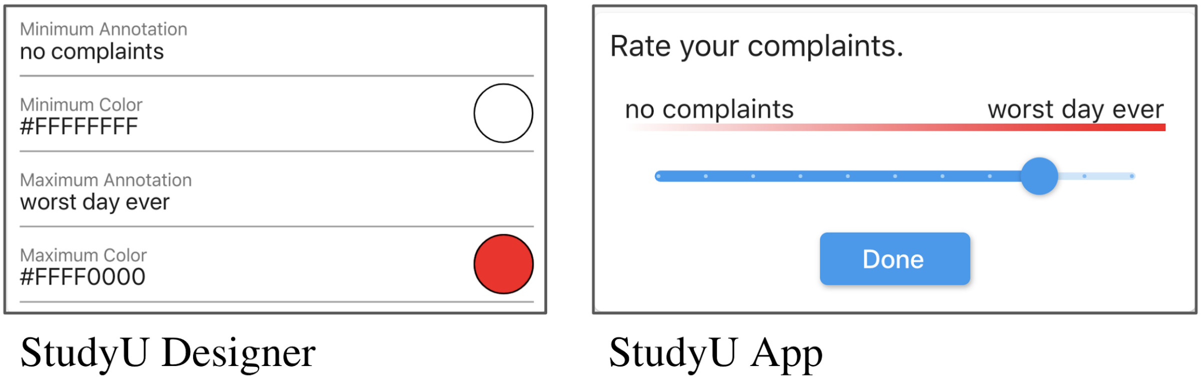

Supplementary Text 5: *StudyU* app

App Onboarding

This part of the app is the only part accessible directly after installing the app. Its purpose is the introduction of the app's objective and of the app's terms and conditions.

Welcome Screen

The welcome screen is the first screen the users reach after opening the app. It is kept clean to not overwhelm the users. The only elements are our app logo, a button to go to the next screen, the terms and conditions, and a button to reach the about screen if users want to get more information about the app and N-of-1 trials before starting a study.

About Screen

The about screen is intended to explain the concept of the app to users in a way that makes them want to participate in a study. Therefore, in our version the users are introduced to the idea of N-of-1 trials by means of an everyday example. In order not to overload the screen with text, single scrollable pages were created, which are rounded off by individual icons for clarification.

Terms and Conditions Screen

This screen displays the terms and conditions the users need to accept in order to use the app. The contents of this screen can be saved to the user's device with a button at the bottom of the list. The file gets created with the currently selected language and saved to Downloads on Android and to a separate folder in Data on iOS. The user can finish the onboarding and reach the study selection if all terms are accepted by clicking the checkboxes.

Study Onboarding

After the user was introduced to the app and the terms of service, the next step is starting a study. This process begins with selecting a study, checking for eligibility and configuring it to the user's interests. The users are also taken to the beginning of this part of the app, after aborting a study selection, after opting out of a study or after finishing a study and starting a new one. During this onboarding, the users can navigate back and forth through the steps with buttons at the bottom of the screen and progress bars how the progress of the onboarding from the eligibility checks onward. This is designed to make the process transparent and keep navigation uniform.

Study Selection Screen

The study selection screen is the start of every study. It displays a list of available studies with their name, a representative icon chosen by the researcher and a short description of the study. After selecting a study, the user can start the study specific onboarding with the study overview screen.

Study Overview Screen

This screen offers an overview over the study the user chose. The main points are the length of the study phases, the (minimum) duration of the study, a description of the study and information about the researchers responsible for the study. This screen has further potential to be extended with additional information the researchers want to provide to the users about their study. There is no interactive element on this screen and the users can continue to the eligibility check questionnaire.

Eligibility Check Screen

The eligibility check is used to prevent users from taking part in a study they are not suitable for. The questions for the check get added to a list one at a time, after the users answered the previous question. This allows the users to focus on one question and also to change an answer to an earlier question in case they made a mistake. When the users correct an answer, the eligibility questions get reset to the corresponding question to keep that step-by-step flow. The questions can be interdependent so that certain questions don't get asked if they are not necessary based on the answer to a previous question. The supported questions are simple yes or no questions or choice questions with either multiple possible selections or a single one. If the users give an answer that disqualifies them from taking part in the study, a popup informs the users about that fact with the given reason and proposes correcting an eventual mistake or going back to the study selection. This is shown in the figure below. If nothing prevents them from taking part in the study, after the last question has been answered the popup will allow the users to continue to the intervention selection.

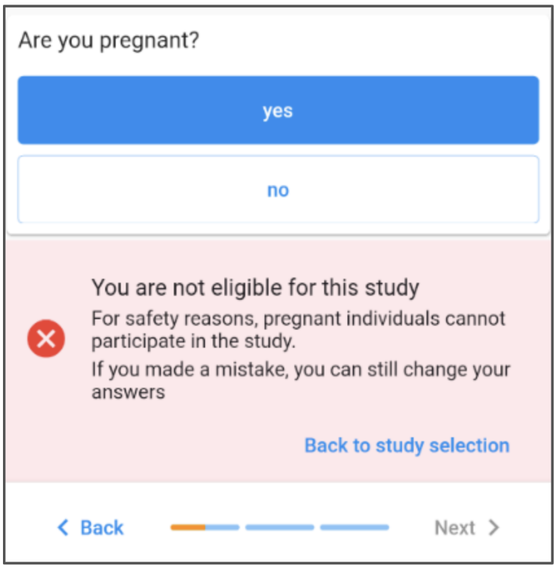

Intervention Selection Screen

This screen allows the users to customize the study according to their interests by choosing two interventions that will be compared during the study. The interventions can be selected from a list defined by the researcher and are presented in a short way with their icon, name and the corresponding daily tasks ordered by time. The users can get further information by tapping an information icon next to the name of the intervention that contains the description

of the intervention. After two interventions have been selected (no more than two interventions can be selected), the users can progress to the journey overview screen.

Journey Overview Screen

The journey overview screen displays the final study schedule of the interventions to the users based on the previously selected interventions and the sequence that was set by the researcher. The starting date is displayed next to each study phase and the last entry of the list is the final date when results will be available. This screen is followed by the consent screen.

Consent Screen

The consent screen is modular in the form of topic-specific boxes. The number and content of the boxes (text, images, animated video) can be defined by the researcher in the *StudyU* Designer. The rationale behind the design of the screen is to make the process of education and consent screen as minimal and at the same time as attractive as possible. Therefore, icons or animations can be added to the boxes and users can recognize which box has already been opened by its color that changes after clicking on it. At the end of the process, users have the opportunity to refuse to participate in a study or to give their consent. The implementation requires users to have read the complete content and agree to it in order to give consent and proceed further. The consent can also be saved on the user's device with a button in the upper right corner of the screen, similar to the terms of service. By choosing to continue, the user finishes the onboarding process and gets redirected to the dashboard to start the study.

Study Participation

The screens of this part of the app are the main part that is accessible during a running study. They allow the user to do the study tasks, look back into the results of past studies and configure some basic app settings. After a study has been started, the app will also send reminders when tasks are supposed to be done as shown below.

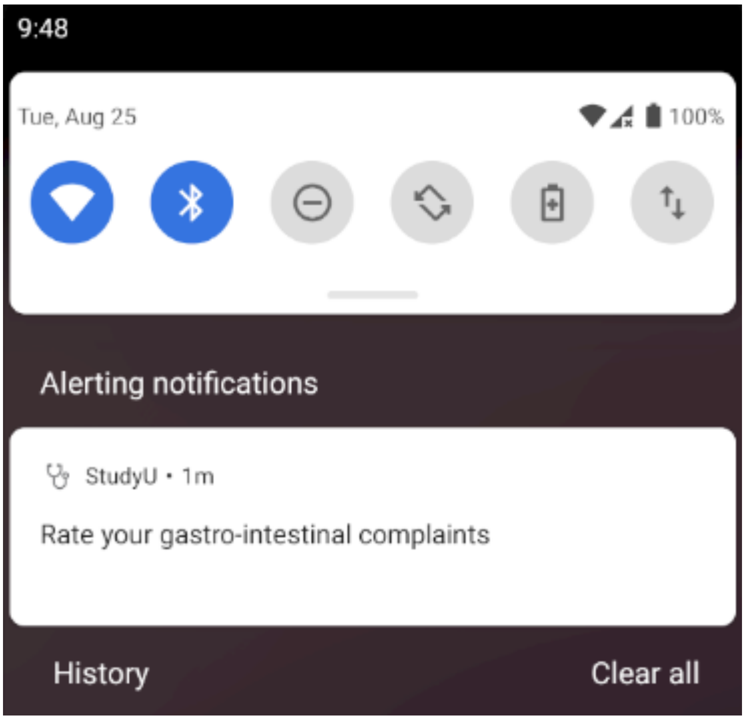

Dashboard Screen

The dashboard screen is the main screen for the user to access the app's functionality. The focus of this screen is the list of tasks the user has to complete to progress with the currently selected study. The tasks are represented with small cards to allow grouping by the time the tasks shall be conducted. The cards only consist of the title of the tasks and a completion status checkbox to allow for a compact overview even if many tasks are present. At the top of the screen is a timeline with the different intervention icons surrounded by indicators showing how many days have been completed during each phase. Below that timeline is the name of the current intervention and next to it is an information icon that contains the interventions description. These elements display the current study state in a concise way. At the top of the screen, the users can reach the contact screen, the report history and the settings.

Task Screen

The task screens are used by the users to complete study tasks. Currently, the two supported task types are checkmark tasks and questionnaire tasks. This aspect of the app is modular and can easily be extended with more task types. The checkmark task screen contains instructions on how to complete the task and a button to confirm completion. The eligibility questions screen contains a list of questions similar to the eligibility check. In addition to the multiple-choice questions and Boolean questions, this eligibility questions also support slider questions that allow the user to rate an aspect on a scale according to their mood. These sliders are either annotated with a color gradient and text or emojis to make the UI visually more appealing. After completing a task, the users are redirected to the dashboard screen.

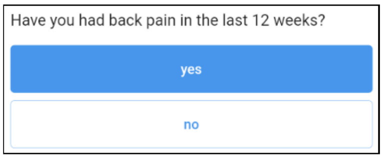

(a) A boolean question.

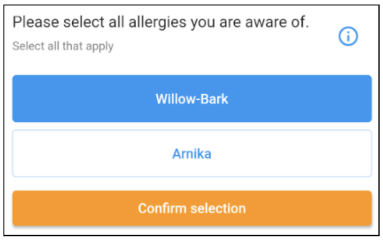

(b) A multiple choice question.

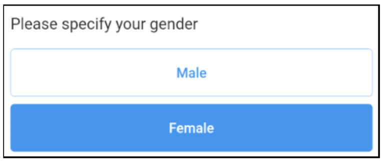

(c) A single choice question.

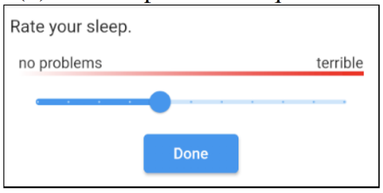

(d) A visual analogue question.

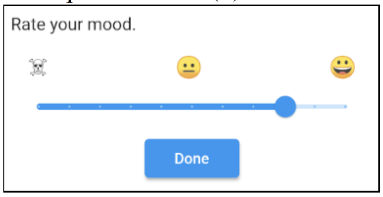

(e) An annotated scale question.

Contact Screen

This screen is a hub for general information concerning the app. It references a Frequently Asked Questions (FAQ) screen with answers to basic user questions, a screen with contact information and the about screen.

Report History Screen

The report history screen displays a list of studies in which the users have participated in the past. Selecting a study name redirects the users to the corresponding report screen.

Report Overview Screen

We assume that knowledge about the previous course of complaints under certain interventions could influence the evaluation of complaints in the further course. Therefore, users should only have access to the results after a certain study period. Users should be able to access the results as soon as they reach the minimum study duration. However, it should be explained to users in advance that more reliable results can be expected the longer they remain participants in the study. We defined countable observations, which include only those that were completed on countable days and on which all adjacent interventions were completed.

Besides the performance report, the report overview includes the report sections defined by the researchers. One of the report types uses a linear regression model to determine which intervention improves the outcome. To achieve this, the desired outcome is used as the dependent variable. The independent variables are two dummy variables indicating whether intervention A or intervention B were active for the sample. The time since the start of the study is included to correct for a linear trend. Values predicted by this model are displayed

with 95% confidence intervals in a bar chart. A hypothesis test is performed whether the regression coefficient of the intervention is zero or not using the large-sample Wald test statistic and a significance level of 0.05. A textual description of the comparison is provided in the report as well.

Performance Screen

Users might wonder why the progress bars are not filling-up, e.g., if they just complete the observations but skip interventions (which would not be countable observations). Therefore, we built the performance details screen that shows for every intervention and observation how many were completed with the aim of reaching the suggested study length.

Settings Screen

This screen allows the user to configure basic app functionality. It displays the active study name and enables them to change the language, opt-out/abort the current study and delete the user data on the device. The currently supported languages are German and English and concern only the UI elements of the app itself. Adding new languages to the app is very simple because the text strings are collected in a single file per language. Opting out of a study deletes the user's progress of the current study and takes them back to the study selection. Deleting the user data removes the user's anonymous account from the device and server and redirects them to the welcome screen. Data for studies that were finished by the users is kept on the server and not deleted.

Supplementary Figures

Supplementary Figure 1: Definition of interventions in the designer and selection of interventions in the app.

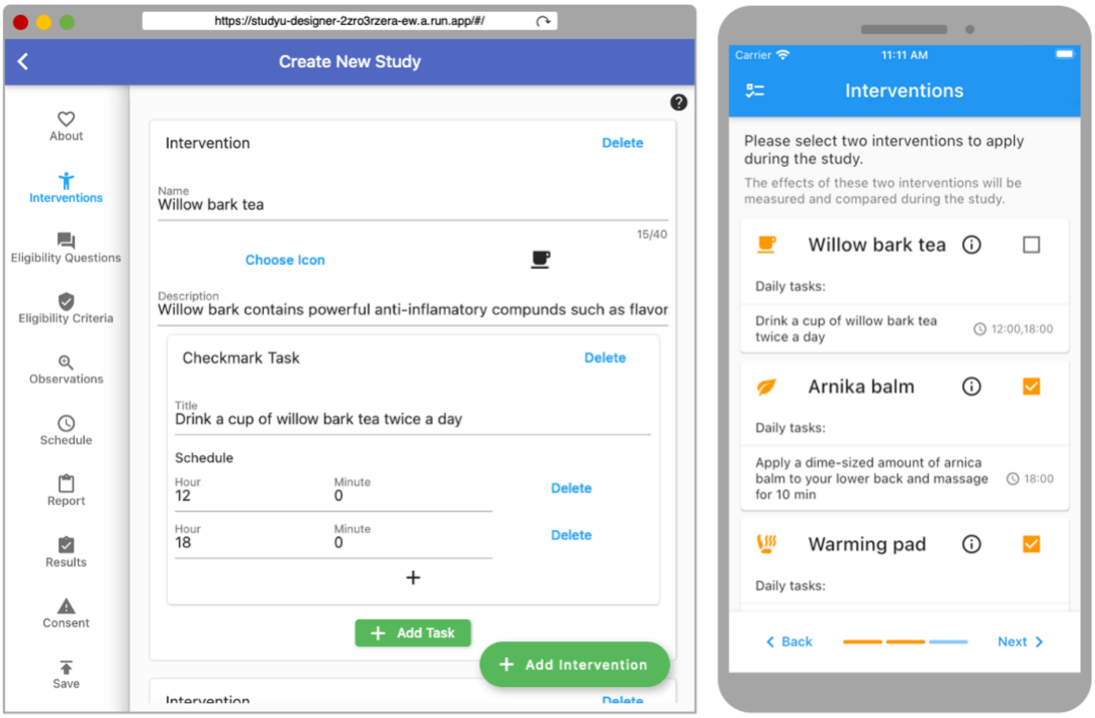

Supplementary Figure 2: Eligibility questions and criteria in the designer and eligibility screen in the app.

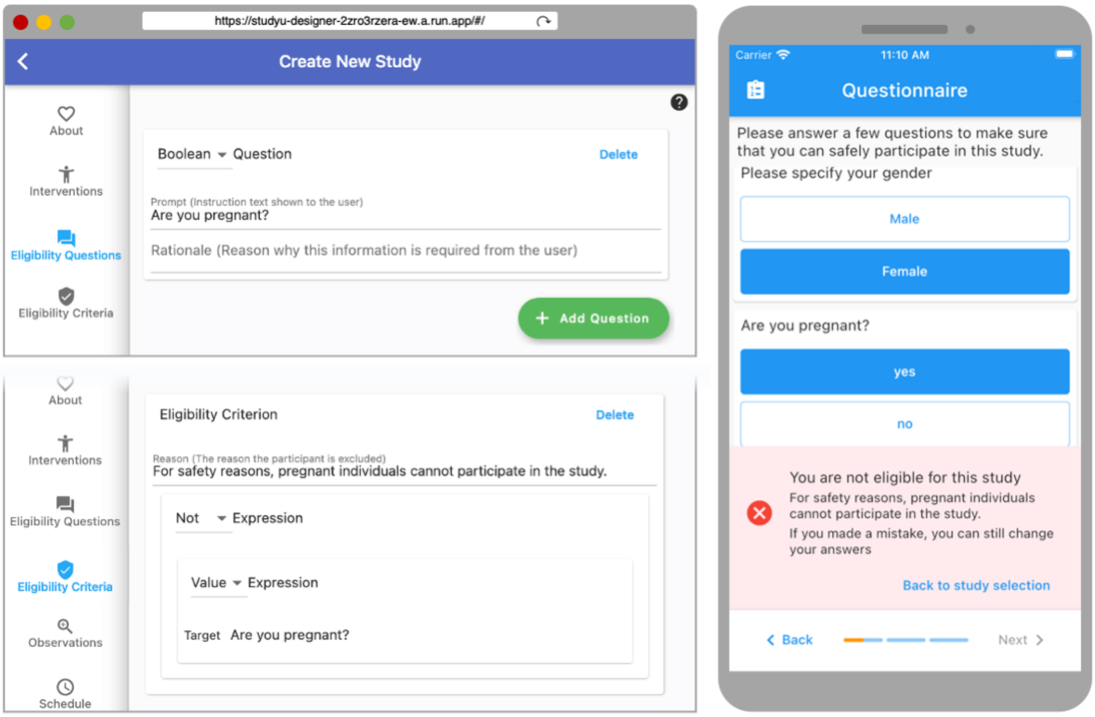

Supplementary Figure 3: Definition of observations in the designer and the screens for the daily overview and outcome reporting in the app. In the example, the participant has to rate the individual pain level and mood on a Likert scale as well as medication intake.

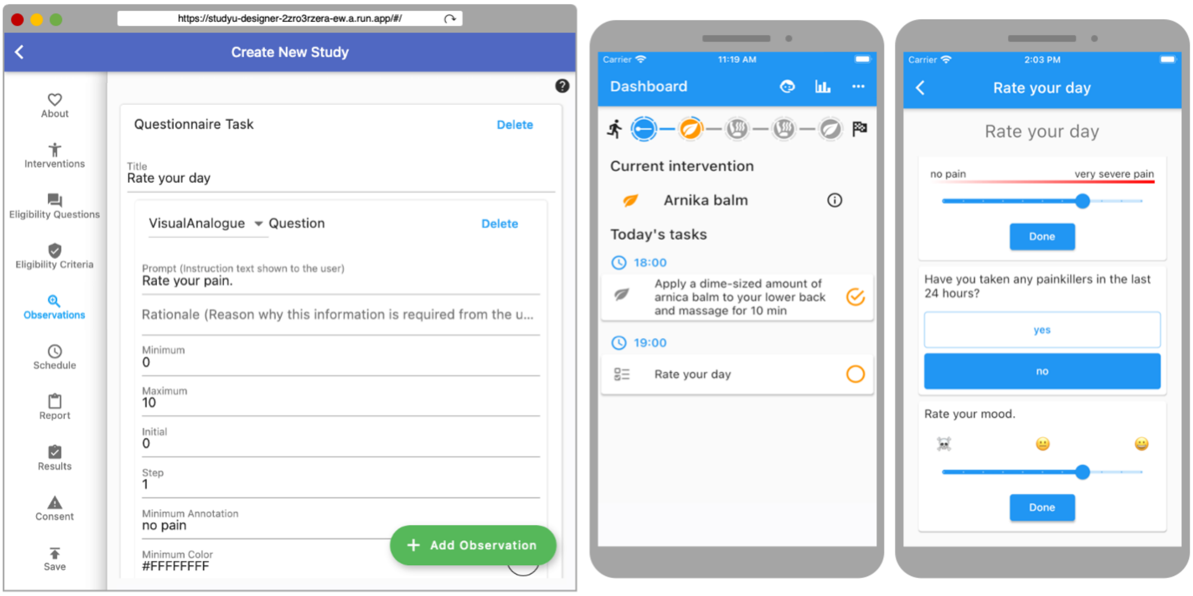

Supplementary Figure 4: Consent definition in the designer and consent screen in the app. When selecting one consent item, details are displayed.

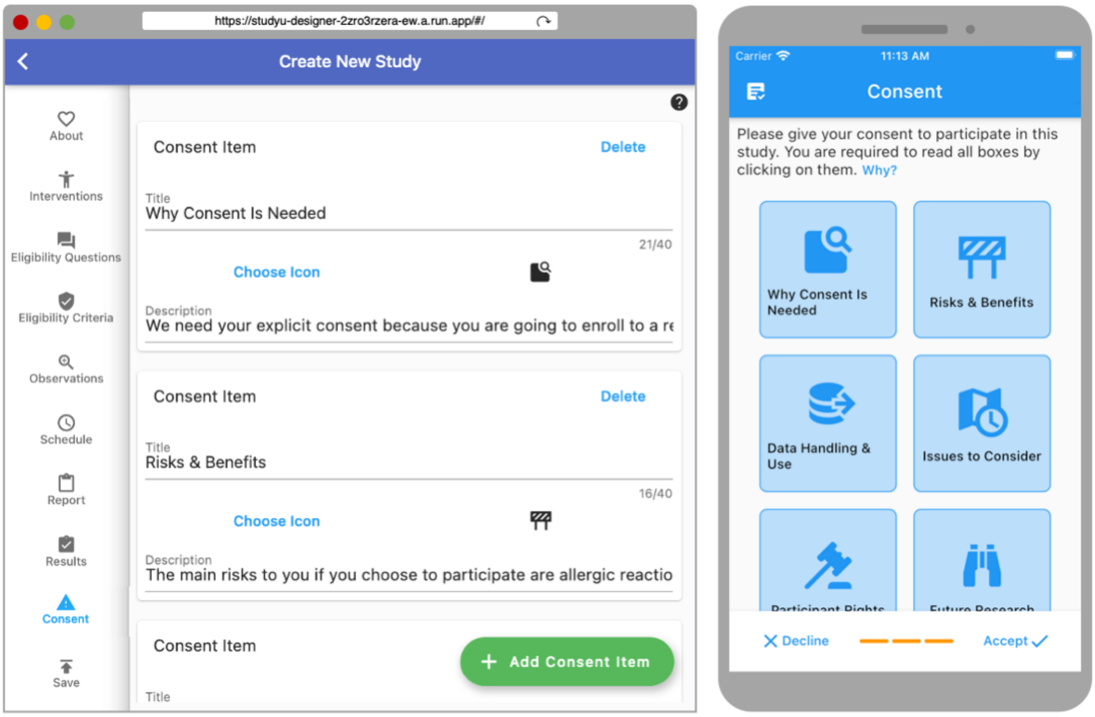

Supplementary Figure 5: Overview of the full StudyU study model. The notation is based on the Unified Modeling Language (UML) class diagram notation: it defines properties of single classes in rectangles and associations between multiple classes as connections. The associations shown in this diagram with a filled diamond at one end mean that one class, e.g., 'Study', is composed of another class, in this case StudyDetails. Numbers shown at associations indicate how many instances of one class take part in this association, e.g., n 'Observation' objects can be associated with one 'StudyDetails' object.

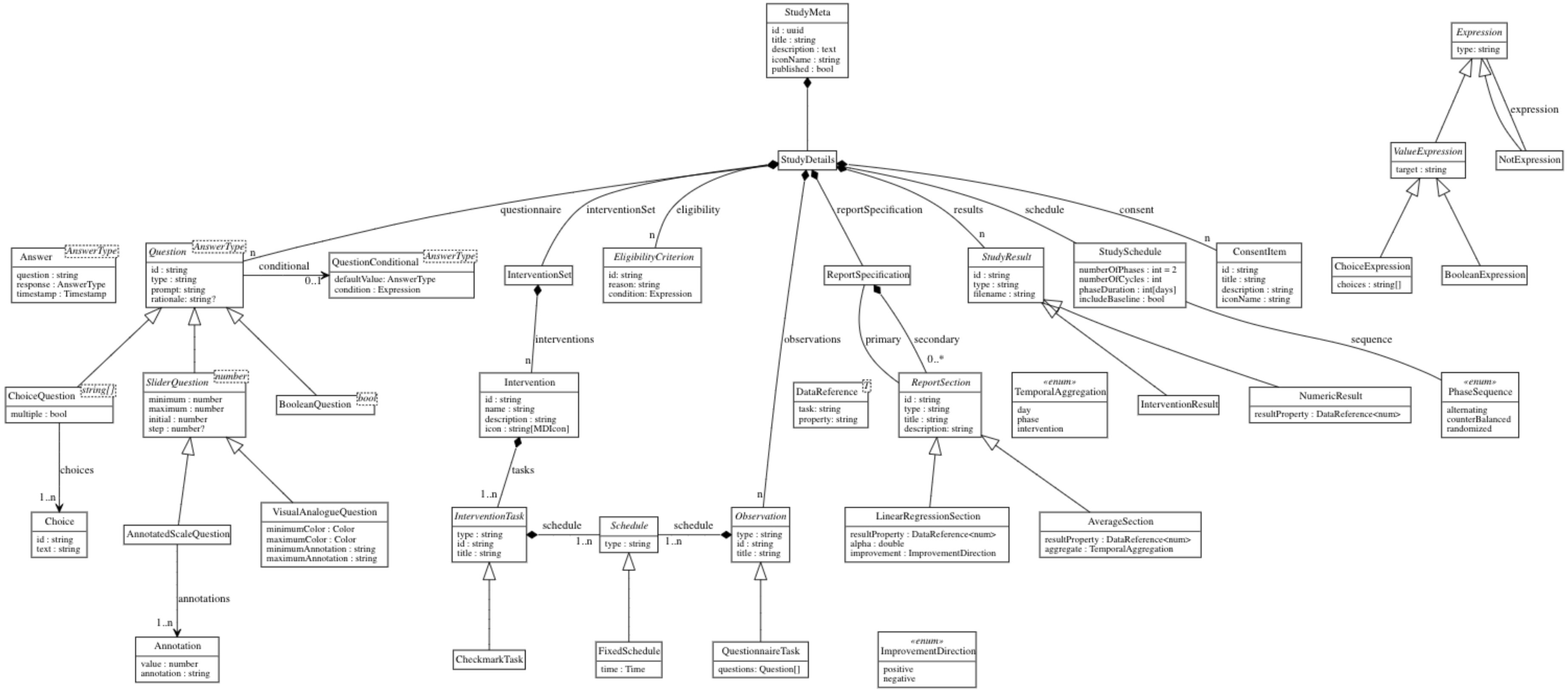

Supplement: Multimedia Appendix 1 [file jmir_v24i7e35884_app1.pdf]
